# *In Vivo* Validation of Simultaneous Non-Contrast Angiography and intraPlaque Hemorrhage (SNAP) Magnetic Resonance Angiography: An Intracranial Artery Study

**DOI:** 10.1371/journal.pone.0149130

**Published:** 2016-02-10

**Authors:** Jinnan Wang, Maobin Guan, Kiyofumi Yamada, Daniel S. Hippe, William S. Kerwin, Chun Yuan, Peter Börnert, Xihai Zhao

**Affiliations:** 1 Clinical Sites Research Program, Philips Research North America, Briarcliff Manor, New York, United States of America; 2 Department of Radiology, Yangzhou First People’s Hospital, Yangzhou, China; 3 Department of Radiology, University of Washington, Seattle, Washington, United States of America; 4 Center for Biomedical Imaging Research, Department of Biomedical Engineering, Tsinghua University, Beijing, China; 5 Tomographic Imaging Systems, Philips Research Europe, Hamburg, Germany; Weill Cornell Medical College, UNITED STATES

## Abstract

**Objectives:**

Simultaneous Non-contrast Angiography and intraPlaque hemorrhage (SNAP) technique was recently proposed for joint MRA and intraplaque hemorrhage (IPH) imaging. The purpose of this study is to validate SNAP’s MRA performance in patients with suspected intracranial artery disease.

**Methods:**

SNAP and time-of-flight (TOF) techniques with matched field of view and resolution were applied on 15 patients with suspected intracranial artery disease. Both techniques were evaluated based on their detection of luminal stenosis of bilateral middle cerebral arteries (MCA) and the delineation of smallest visible branches (SVB) of the MCA. Statistical analysis was conducted on the artery level.

**Results:**

The SNAP MRA was found to provide similar stenosis detection performance when compared with TOF (Cohen’s κ 0.79; 95% Confidence Interval: 0.56–0.99). For the SVB comparison, SNAP was found to provide significantly better small artery delineation than TOF (p = 0.017). Inter-reader reproducibility for both measurements on SNAP was over 0.7. SNAP also detected IPH lesions on 13% of the patients.

**Conclusions:**

The SNAP technique’s MRA performance was optimized and compared against TOF for intracranial artery atherosclerosis imaging and was found to provide comparable stenosis detection accuracy. Along with its IPH detection capability, SNAP holds the potential to become a first-line screening tool for high risk intracranial atherosclerosis disease evaluation.

## Introduction

Both luminal stenosis and intraplaque hemorrhage (IPH) have been identified as key imaging biomarkers of atherosclerotic disease which can be used to stratify patients by the risk of developing ischemic cardiovascular events. Luminal stenosis is widely adopted in clinics as a surrogate marker of the disease for clinical decision-making [1,2]; on the other hand, as an emerging biomarker, IPH occurred in extracranial carotid arteries and intracranial arteries has been demonstrated to be associated with increased risk for clinical events [3–5]. Additionally, it is well evidenced that the IPH will stimulate the progression of the carotid plaques [6,7]. An imaging technique that can reliably detect both biomarkers suggests significant clinical potential.

The Simultaneous Non-contrast Angiography and intraPlaque imaging (SNAP) sequence was recently proposed as a candidate technique to address this need [8]. The SNAP approach can provide a full 3D luminal MRA and a naturally registered 3D IPH image in a single acquisition. Theoretical analysis indicates that SNAP is more sensitive to IPH than any existing imaging techniques [8]. When compared with established techniques in carotid artery imaging, SNAP was found to provide comparable lumen size measurements and IPH detection rates [8].

Intracranial atherosclerosis disease is a significant but often overlooked contributor to ischemic strokes. Previous reports suggest that it represents 9–15% of all the ischemic strokes in US [9]and that the prevalence can be even higher, sometimes even over 50% [10,11], in certain racial groups [12]. Intracranial atherosclerosis has even been suggested as one of the most common causes of stroke worldwide [13]. It is therefore clinically meaningful to explore whether the combination of luminal stenosis and IPH can serve as a better marker for intracranial diseases as well.

Although SNAP has been used to image intracranial arteries, the luminal stenosis detection accuracy of SNAP has not been validated in any vascular beds. The aim of this study is to evaluate SNAP MRA performance by comparing with the clinically more commonly used Time-of-Flight (TOF) technique on a group of patients with clinically suspected intracranial disease. In addition to comparing luminal stenosis of major arteries, the capability of SNAP MRA to resolve small arteries will also be compared with that of TOF.

## Materials and Methods

### Study population

Three healthy volunteers (all male, age range: 26–33) were recruited for sequence optimization. Subsequently, for sequence validation, 15 consecutive patients with suspected intracranial disease and agreement to participate in the study were recruited. The study protocol was approved by the institutional review board of Tsinghua University School of Medicine and the written informed consents were obtained from all subjects.

### Sequence optimization

The SNAP sequence [8] is an inversion recovery magnetization prepared gradient echo sequence, followed by a series of low flip angle acquisition serving as the reference. The reference acquisition is essential for phase-sensitive reconstruction [14]. SNAP MRA is generated based on the naturally long T_1_ property of the blood: once inverted, it typically takes much longer time for blood to relax back towards thermal equilibrium compared to other tissues. Consequently, when imaged at the proper TI, blood will be the only tissue that remains negative in the phase-sensitive images. This allows the generation of an ideal MRA image when only the negative magnetization is visualized. As detailed in the original papers [8,15], two conditions are usually required to achieve SNAP MRA with reasonable quality: the blood is properly inverted and the acquisition time is properly selected.

For the latter condition, assuming all the key tissues involved in intracranial artery imaging (blood in the lumen, IPH, and vessel wall) possess similar T_1_ relaxation times as in the carotid arteries, the optimal image contrast for carotid artery was considered reasonable for intracranial arteries. Therefore, the TI and inversion recovery interval times were not further optimized in this study.

To make sure blood is properly inverted, the original SNAP paper proposed two criteria to optimize the inversion pulse coverage in the foot-head direction based on estimated upper/lower boundary of flow velocity [15]. For the carotid artery, it is relatively straightforward to estimate the upper and lower boundary of blood velocity along the inversion direction as the overall flow direction is uniform. However, given the tortuous nature of intracranial arteries and the heterogeneous flow direction in the region, it would be difficult to estimate inversion coverage using the same criteria. In vivo optimization of the inversion coverage is therefore needed to adequately optimize SNAP for intracranial artery imaging.

For in vivo optimization, a whole body 3T scanner (Philips Achieva R3.21, Best, the Netherlands) and an 8-channel phased-array brain coil were used for image acquisition. After scout scans, a set of axial SNAP images were acquired around the MCA section. Among the SNAP acquisitions, all imaging parameters were the same except the inversion slab thickness. Slab thicknesses of 150mm, 300mm and 450mm were considered, as was a non-selective excitation. The rest of the imaging parameters were: Phase-sensitive Inversion-recovery enabled 3D inversion recovery turbo field echo, inversion recovery interval time 1970ms, TR/TE 10/5.5ms, flip angle 11°, FOV 160×160×50mm^3^, acquired matrix size 1.2×1.2×1.2mm^3^, interpolated to 0.6×0.6×0.6mm^3^, SENSE factor 2, scan time 1min06sec.

As the inversion slab thickness has no impact on the IPH contrast, MRA quality will be the sole point of comparison for parameter selection. The thickness which presented the highest MRA quality was selected as the optimal imaging parameter.

### Validation

Multi-slab TOF MRA was used as the reference technique for SNAP validation as it has been commonly used in clinical intracranial scans due to its robust performance in the region [16]. Furthermore, its acquisition time is not limited by the first pass duration, so a relatively high imaging resolution can be achieved, and it is a non-contrast MRA technique like SNAP.

In consideration of the scanning efficiency, only a thin slab of a multi-slab TOF protocol was used in this study. By using only one thin slab, the scan time can be substantially reduced without compromising the image quality. The same imaging hardware was used as in the sequence optimization study. The imaging parameters for SNAP were: Phase-sensitive Inversion-recovery enabled 3D inversion recovery turbo field echo, inversion recovery interval time 1970ms, TR/TE 10/5.5ms, flip angle 11°, FOV 160×160×50mm^3^, acquired matrix size 1×1×1mm^3^, interpolated to 0.5×0.5×0.5mm^3^, scan time 2min40sec. The optimal inversion slab thickness identified in the previous section was used. The geometric parameters for TOF were identical to SNAP and the other parameters were: 3D fast field echo, TR/TE 26/3.5ms, flip angle 20°, scan time 1min40sec. Matched SNAP and TOF imaging volumes were placed centered at the MCA arteries in the foot-head direction. Due to the thin slab coverage, only the MCA MRA was evaluated.

### Image analysis

After the SNAP images were obtained, they were first processed to realize the traditional 3D maximum intensity projection (MIP) MRA view as previously described [8].

A neuroradiologist with over 5 years of experience in neuroradiology reviewed all images. To ensure a fair comparison, all images were grouped into two sets: one set contained a randomly selected image (SNAP or TOF) from each subject; the other set contained the other images from the same subjects. The number of SNAP and TOF images was balanced between the two sets and each image was given a random number so the paired SNAP and TOF images could not be linked during review. The two sets of images were reviewed separately with a 2-week interval in between to avoid memory bias. Both the left and right MCA were analyzed in every image.

To evaluate luminal stenosis detection accuracy, the M1 segment of each MCA was assessed by the neuroradiologist. Stenosis was defined as segmental reduction of lumen with a residual flow or signal loss on MIP images; otherwise the artery will be deemed normal [17]. The severity of stenosis measurement is heavily impacted by the window/level settings. To ensure a fair comparison, two dataset need to have identical settings. This is however impossible to achieve in our study. SNAP images take the advantage of doubled dynamic ranges from phase sensitive images. SNAP MRA, in particular, was reconstructed from negative signals from phase sensitive images, which is drastically from the more traditional magnitude image based TOF images. Through our testing implementation in getting a comparable window/level settings between SNAP and TOF, we found a bias will invariably introduced between the two techniques no matter how the levels were set. We therefore decided to compare only the existence of stenosis in our study.

**Fig 1:**
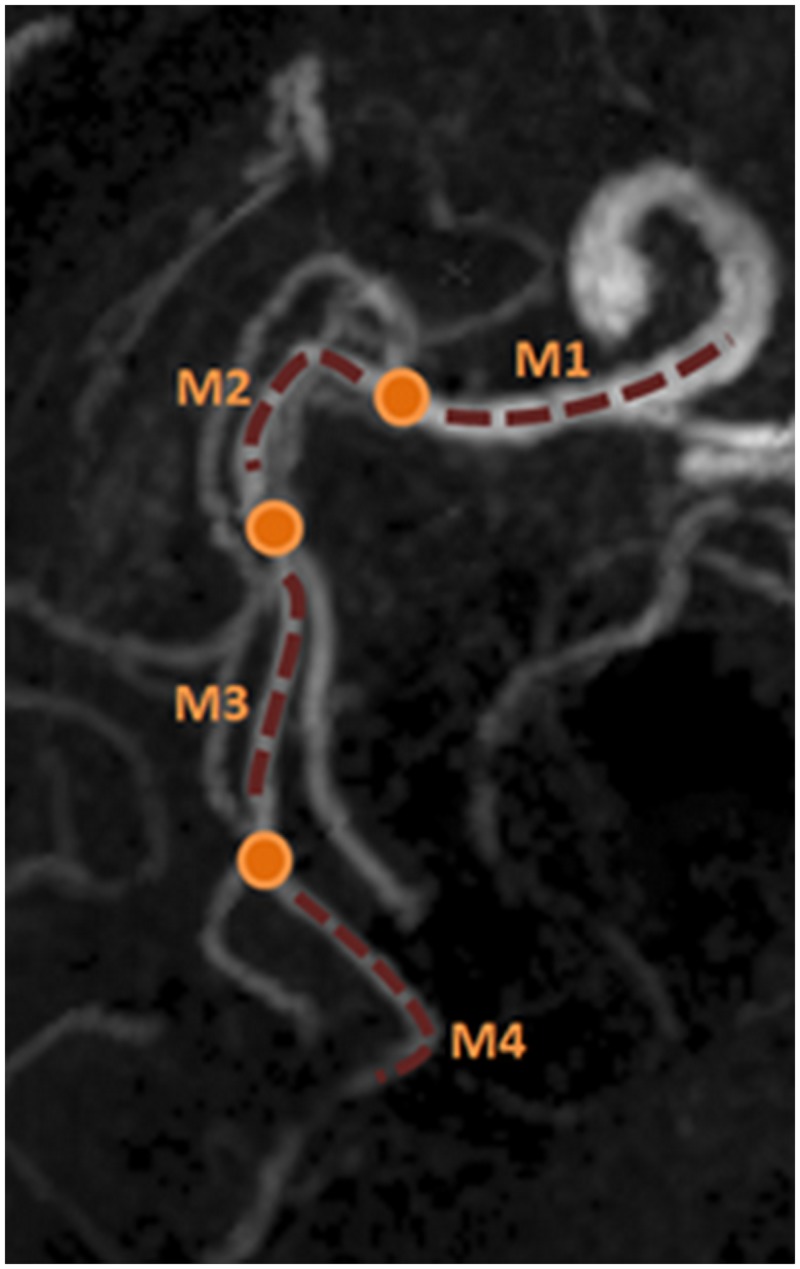
Definition of MCA segments for SVB comparison. The orange dots identify the bifurcations and the dotted lines delineate the different segments (M1-M4) of the MCA artery. Note that the oblique projection direction is used to better visualize the different segments.

To evaluate the capability of SNAP to resolve small arteries, the Smallest Visible Branch (SVB) of the MCA artery was also recorded by the radiologist. In this comparison, the smallest branch that could still be fully delineated on the MR image was defined as the SVB of this particular artery. To facilitate the comparison, MCA segments were named as M1- M4 based on the established method [18]. It was also demonstrated in Fig 1.

The presence or absence of intraplaque hemorrhage and/or thrombosis was also evaluated on all subjects using dual-contrast MIP images that can simultaneously show luminal MRA and IPH and thrombosis (IPH/T). IPH/T was not differentiated in this study due to the finer caliber of intracranial arteries and the spatial resolution achievable in a reasonable scan time. The presence of IPH/T was defined as a hyperintensive red (positive) signal as described in [8].

### Statistical analysis

Luminal stenosis comparison between SNAP and TOF was evaluated by Cohen’s Kappa (κ), and the SVB comparison was evaluated by Wilcoxon’s signed-rank test. Dependence between arteries from the same subject was accounted for in two ways. A 95% confidence interval for κ was computed using the non-parametric bootstrap, where resampling was done by subject [19]. The Wilcoxon signed-rank test was adapted to handle paired arteries by implementing a permutation test where the arteries from the same subject were always permuted together [19]. A p value of <0.05 was considered as statistically significant. All statistical analysis was conducted by SPSS 15.0 (SPSS Inc, Chicago, IL, USA).

## Results

### Sequence optimization

**Fig 2:**
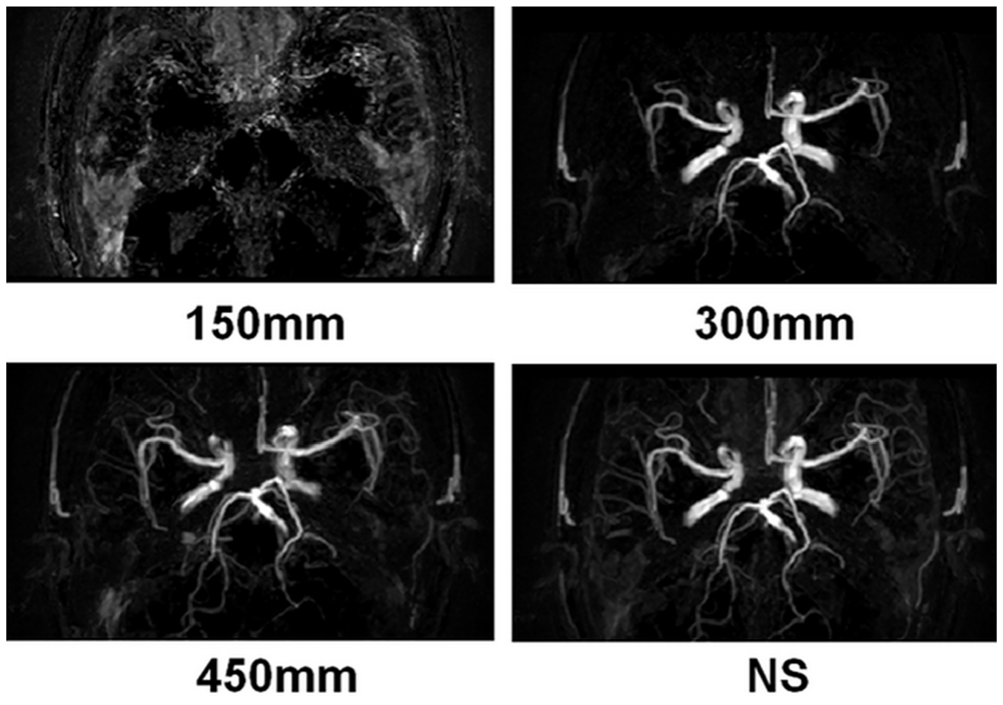
Optimization of inversion slab thickness for SNAP imaging. It is clear that the SNAP MRA performance is best when the inversion pulse was non-selective (NS) compared to the inversion slab thicknesses considered. This implementation was used for the subsequent validation scans.

The performance of SNAP MRA was found to vary by the inversion slab thickness (Fig 2). A non-selective inversion provided the optimal MRA view among all parameters: more small-branches can be visualized (arrows). This may have been due to two characteristics of the sequence: non-selective inversion pulses allow for more complete excitation and the long delay between inversion pulses avoids severe signal saturation. As a result, the non-selective inversion pulse was used for the subsequent validation scans.

### In vivo validation

A total of 15 patients were recruited for the validation study. Both TOF and SNAP were successfully acquired in all patients and the image quality of all images was found to be satisfactory for review.

**Fig 3:**
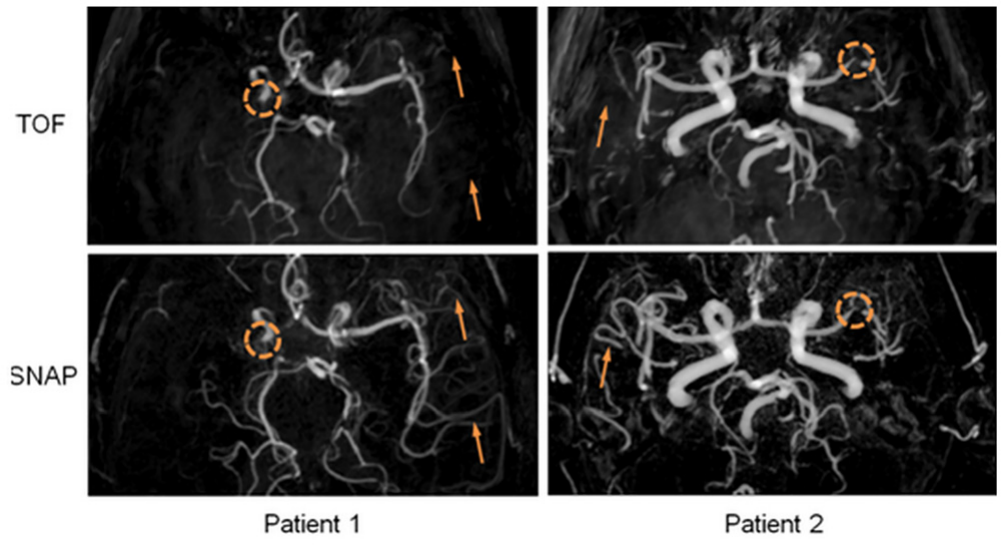
Visual comparison between SNAP and TOF MRA on the same subjects. SNAP and TOF make same detection on stenotic lesions on both subjects (dotted circles). The small artery visualization on SNAP MRA is significantly improved (arrows).

Compared to TOF, 3D SNAP MRA was found to provide improved visualization of the intracranial artery vascular tree, particularly on smaller branches (Fig 3). This was consistently observed across the group.

Of the 30 arteries reviewed, M1 stenosis was identified in 18 (60%) arteries by SNAP and 19 (63%) arteries by TOF. SNAP and TOF agreed on the presence of stenosis in 17 arteries and disagreed on three arteries, resulting in excellent agreement (Cohen’s κ = 0.79; 95% Confidence Interval: 0.56–0.99). One stenotic artery was identified as a total occlusion by both SNAP and TOF.

**Fig 4:**
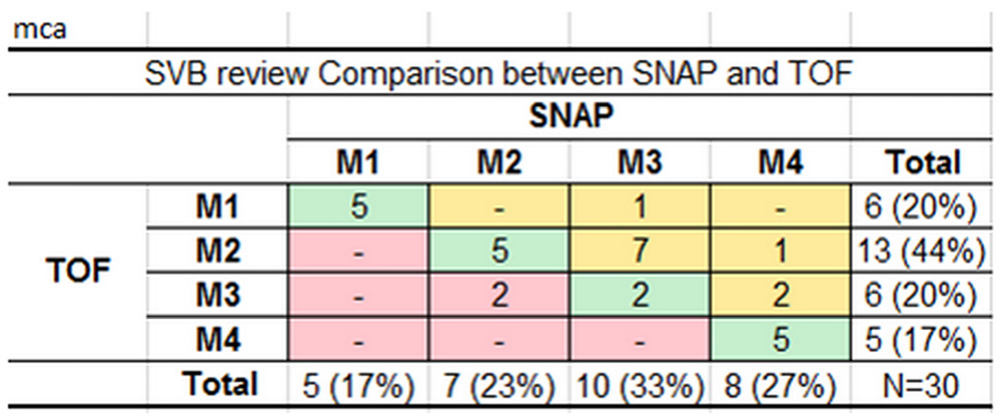
Cross-tabulation of MCA smallest visible branch (SVB) review results by SNAP and TOF MRA. Rows show the number of arteries with the corresponding SVB by TOF and columns show the number of arteries with the corresponding SVB by SNAP. A dash indicates that no arteries had that pair of results. The diagonal shows the number of arteries with the same SVB on both SNAP and TOF (green), while the cells above (yellow) or below (red) the diagonal show the number of arteries where SNAP or TOF was better than the other, respectively. SNAP visualizes more distal branches than TOF in 11 arteries, less in 2 arteries and performs equally in the remaining 17 arteries.

A comparison of the smallest visible branch (SVB) review results by SNAP and TOF is shown in Fig 4. The most common SVB was M3 for SNAP (33% of arteries) and M2 for TOF (44% of arteries). Five arteries with SVB = M1 were identified because the M1 segments were found to be occluded on both techniques. SNAP was able to visualize a more distal branch than TOF in 11 (37%) arteries and less distal in 2 arteries (7%) while the SVB was the same for both in the remaining 17 arteries. Statistically, SNAP was found to visualize significantly more branches than TOF (p = 0.017).

**Fig 5:**
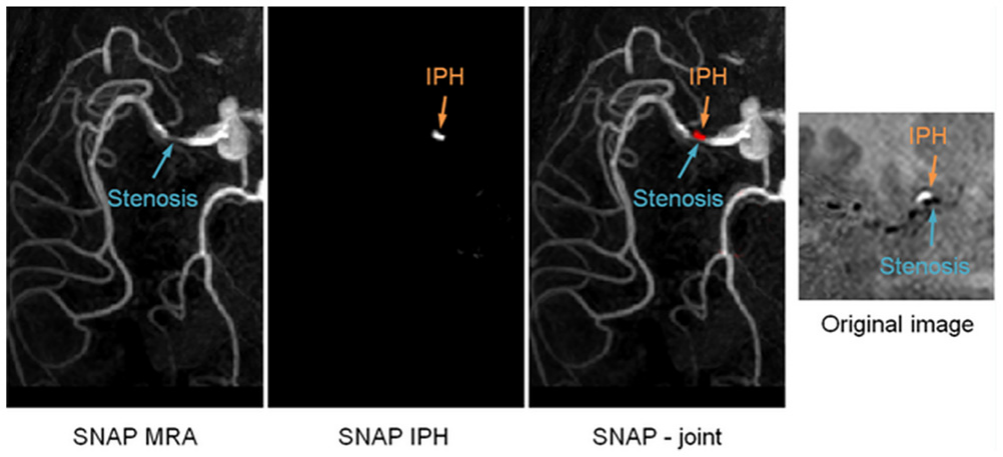
The simultaneous visualization of MRA and IPH/Thrombosis using the SNAP sequence. On the left are reformatted SNAP MRA, IPH/T and joint MIP view to facilitate image review. One the right is the original image at the cross-section of the MCA artery. Obvious contrast between IPH/Thrombosis (positive magnetization) and luminal (negative magnetization) signal can be found.

It is also noteworthy that 3 IPH/T lesions were identified on 2 patients, giving an observed incidence rate of ~13% in this patient population. Using the dual-MIP display method described before [8], a color-coded 3D SNAP image allows for easy simultaneous visualization of both luminal stenosis and IPH in the same review session. An example was shown in Fig 5.

## Discussion

The SNAP sequence has potential for future clinical evaluation of atherosclerotic disease due to the simultaneous MRA and IPH/T visualization obtained in one scan. The sequence was originally developed and tested in the carotid artery [8]. In this study, the SNAP sequence was optimized for intracranial artery imaging and, for the first time, validated for luminal stenosis detection accuracy against an established technique of TOF.

SNAP MRA’s improved visualization of smaller arteries was demonstrated in this study, both qualitatively and quantitatively. This improvement is likely caused by two factors: the improved background suppression and the reduced dependence on flow velocity. In terms of background suppression, as explained in the ‘Materials and Methods’ section, the SNAP technique is optimized to maintain negative magnetization only for blood. In the SNAP MRA view, all the other tissues are automatically masked so that theoretically, complete background suppression can be achieved. In TOF, on the other hand, unsuppressed tissues like fat contribute to a significant portion of the background signal, making the visualization of smaller arteries more challenging. In terms of reduced reliance on flow velocity, any incoming blood that flows into the FOV between two inversion pulses (1.97 seconds) can be properly imaged in SNAP MRA while TOF MRA usually saturates after ~10 TR (depending on the parameters used), which corresponds to roughly 0.26 seconds in our study. This much longer in-flow time also makes SNAP a more robust technique in visualizing smaller arteries.

The incidence of intracranial IPH has only been occasionally reported in the literature [4,20–22], which may be due to a possibly low incidence rate and/or the lack of suitable imaging techniques. The assessments of IPH by MR T1 imaging in intracranial arteries remain uncertain until a histology validation study was performed recently [23]. The T1 weighted image derived from SNAP imaging makes it possible to detect intracranial IPH. Compared to other IPH detection techniques, SNAP provides the highest sensitivity because of the much improved dynamic range offered by the phase-sensitive reconstruction technique [8]. In this study population, a 13% IPH incidence rate was identified by the SNAP sequence. Our findings are in line with previous reports of 19.6% prevalence in symptomatic and 3.2% in asymptomatic populations [4]. As IPH has been reported as a high-risk feature in carotid artery disease, its clinical risk in intracranial atherosclerosis needs to be better understood. SNAP may become a powerful tool in detecting intracranial IPH.

Contrast enhanced MRA and CT angiography are commonly regarded as more robust than non-contrast MRA techniques in non-invasively detecting luminal stenosis. However, in this study, contrast enhanced MRA and CTA were not used for comparison as they are less commonly prescribed in our center because Gd-based and iodine-based contrast agents make contrast enhanced MRA and CTA less favorable for patients with potentially impaired renal function. TOF MRA is often used as a non-contrast alternative technique for intracranial MRA imaging. Its performance has repeatedly been found to be sufficient in this region [24,25]. In this study, in an effort to reduce the overall imaging time, only a thin slab of imaging volume was used for both SNAP and TOF to ensure a fair comparison. The overall findings are expected to be the same should a multi-slab protocol be adopted for both techniques.

The stenosis detection comparison in this study focused only on lesions in the M1 segments of intracranial arteries. This was intentional as to avoid potential detection discrepancies caused by the small artery visualization difference between the two techniques. As shown in the SVB comparison, TOF may provide suboptimal characterization for arteries in M2-M4 segments, which can confound stenosis detection in those distal segments.

This study has several limitations. First, TOF MRA was used for validation of SNAP imaging in evaluation of intracranial artery stenosis. Clinically, catheter angiography is the gold standard in measuring luminal stenosis of intracranial arteries. Validation of SNAP imaging by catheter angiography in future studies is warranted. Second, the relatively low spatial resolution (1×1×1 mm^3^, interpolated to 0.5×0.5×0.5 mm^3^) used by both SNAP and TOF, as higher resolution images are more commonly used in clinical scans. The implementation of a higher resolution SNAP would require acquisition bandwidth that cannot be afforded by the current gradient systems. Improved receiver technology can potentially help improve the resolution achievable by SNAP without compromising image contrast. This resolution challenge, however, is not expected to impact the findings of this study as SNAP and TOF are still compared at exactly matched scan geometries. Similar findings are expected should higher resolution protocols be adopted by both techniques.

In conclusion, the SNAP technique’s MRA performance was optimized and compared against TOF for intracranial artery atherosclerosis imaging and was found to provide comparable stenosis detection accuracy. The combined high stenosis detection accuracy and high sensitivity to intraplaque hemorrhage lesions paves the way for SNAP’s potential clinical application in intracranial artery imaging.
